# Phase 2 results of idecabtagene vicleucel (ide-cel, bb2121) in Japanese patients with relapsed and refractory multiple myeloma

**DOI:** 10.1007/s12185-023-03538-6

**Published:** 2023-01-24

**Authors:** Daisuke Minakata, Tadao Ishida, Kiyoshi Ando, Rikio Suzuki, Junji Tanaka, Shotaro Hagiwara, Revathi Ananthakrishnan, Shigeki Kuwayama, Mitsufumi Nishio, Yoshinobu Kanda, Kenshi Suzuki

**Affiliations:** 1 Division of Hematology, Department of Medicine, Jichi Medical University, 3311-1 Yakushiji, Shimotsuke, Tochigi 329-0498 Japan; 2 Department of Hematology, Japanese Red Cross Medical Center, Tokyo, Japan; 3 Department of Hematology and Oncology, Tokai University School of Medicine, Isehara, Japan; 4 Department of Hematology, Tokyo Women’s Medical University, Tokyo, Japan; 5 Faculty of Medicine, University of Tsukuba, Ibaraki, Japan; 6 Bristol Myers Squibb, Princeton, NJ USA; 7 Bristol Myers Squibb K.K, Tokyo, Japan

**Keywords:** Relapsed/refractory multiple myeloma, CAR T cell therapy, Japanese patients, Idecabtagene-vicleucel, Triple-class exposed

## Abstract

**Background:**

In the phase 2 KarMMa trial, patients with relapsed/refractory multiple myeloma (RRMM) achieved deep and durable responses with idecabtagene vicleucel (ide-cel), a B-cell maturation antigen-directed chimeric antigen receptor (CAR) T cell therapy. Here we report a sub-analysis of the Japanese cohort of KarMMa.

**Methods:**

Adult patients with RRMM who had received  ≥ 3 prior treatment regimens, including a proteasome inhibitor, an immunomodulatory agent, and an anti-CD38 antibody, and had disease refractory to last treatment received ide-cel at a target dose of 450 × 10^6^ CAR positive T cells.

**Results:**

Nine patients were treated with ide-cel. The overall response rate was 89% (median follow-up, 12.9 months). The best overall response was stringent complete response in 5 patients (56%), very good partial response in 3 (33%), and stable disease in 1. Median duration of response was not reached. All patients experienced grade ≤ 2 cytokine release syndrome and one patient experienced grade 2 neurotoxicity, but all resolved. Two patients died, one each from plasma cell myeloma and general health deterioration.

**Conclusion:**

Ide-cel yielded deep, durable responses with a tolerable and predictable safety profile in Japanese patients with RRMM. These results are similar to those of the non-Japanese population in KarMMa.

**Supplementary Information:**

The online version contains supplementary material available at 10.1007/s12185-023-03538-6.

## Introduction

Despite recent advancements in therapy, multiple myeloma (MM) is an incurable disease with the majority of patients relapsing after first-line therapy with immunomodulatory drugs, proteasome inhibitors (PIs), or anti-CD38 antibodies [1, 2]. The outcomes for patients with relapsed and refractory MM (RRMM) receiving subsequent treatment are poor, with overall response rates (ORRs) of 26–32%, a median progression-free survival (PFS) of 3–5 months, and a median overall survival (OS) of 9–15 months [1, 3–6]. Recently, the advent of chimeric antigen receptor (CAR) T cell therapies has improved outcomes for patients with RRMM [7].

Idecabtagene vicleucel (ide-cel, bb2121) is a B-cell maturation antigen (BCMA)-directed CAR T cell therapy. In the pivotal phase 2 KarMMa trial (NCT03361748), ide-cel demonstrated frequent, deep, and durable responses in triple-class–exposed patients with RRMM [8]. In patients outside of Japan (*N* = 128), at a median follow-up of 24.8 months, the ORR was 73%, with a complete response (CR) rate of 33% and median OS of 24.8 months across all target dose levels (150, 300, and 450 × 10^6^ CAR-positive [CAR+] T cells) [9]. The most common any-grade toxicities were neutropenia (91%) and cytokine release syndrome (CRS; 84%, 4% grade  ≥ 3). CRS was treated with tocilizumab in 67 patients and steroids in 19 patients. Neurotoxicity was reported in 23 (18%) patients, with 5 (4%) grade ≥ 3 events, and was treated with tocilizumab in 3 patients and steroids in 10 patients [9]. At the highest dose of 450 × 10^6^ CAR+ T cells, the ORR was 81%, the CR rate was 39%, and the median OS was 24.8 months [10]. Based on these results, ide-cel was approved for patients with  ≥ 4 prior lines of therapy in the USA (at a target dose of 300–460 × 10^6^ CAR+ T cells) or  ≥ 3 prior lines of therapy in the EU (target dose of 420 × 10^6^ CAR+ T cells) and Japan (target dose of 450 × 10^6^ CAR+ T cells), including an immunomodulatory agent, a PI, and an anti-CD38 antibody [11–13]. In addition, in a retrospective analysis that included patients from the KarMMa trial, significant improvements with ide-cel compared with other MM therapies (immunomodulatory agent, PIs, and anti-CD38 antibodies) were seen in ORR (76.4% vs 32.2%; *p* < 0.0001), PFS (11.6 vs 3.5 months; *p* = 0.0004), and OS (20.2 vs 14.7 months; *p* = 0.0006) [14]. This manuscript reports the results of a sub-analysis of Japanese patients from the KarMMa trial.

## Materials and methods

### Study design and patients

**Fig. 1:**
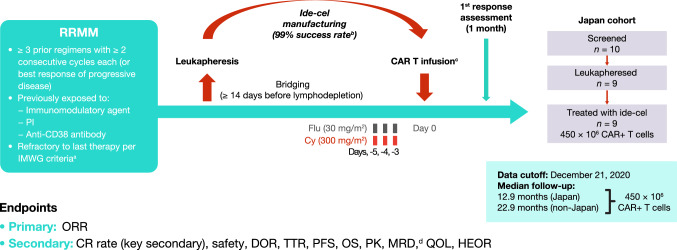
Study design and patient disposition. *CAR* chimeric antigen receptor, *CR* complete response, *Cy* cyclophosphamide, *DOR* duration of response, *Flu* fludarabine, *HEOR* health economics and outcomes research, *ide-cel* idecabtagene vicleucel, *IMWG* International Myeloma Working Group, *MRD* minimal residual disease, *ORR* overall response rate, *OS* overall survival, *PFS* progression-free survival, *PI* proteasome inhibitor, *PK* pharmacokinetics, *QOL* quality of life, *RRMM* relapsed/refractory multiple myeloma, *TTR* time to response. **a** Defined as documented disease progression during or within 60 days from last dose of prior antimyeloma regimen. **b** Based on 1 manufacturing failure in the non-Japan cohort out of 149 patients in Japan and non-Japan cohorts who underwent leukapheresis (target dose levels 150–450 × 10^6^ CAR+ T cells). **c** Hospitalization for 14 days was required after infusion. **d** By next-generation sequencing

Details on the full study design and eligibility criteria have been reported by Munshi et al., 2021 [8]. Briefly, key inclusion criteria included patients aged  ≥ 18 years who had received  ≥ 3 prior regimens (including an immunomodulatory agent, a PI, and an anti-CD38 antibody) with  ≥ 2 consecutive cycles each unless progressive disease (PD) was the best response, and had disease refractory to last treatment regimen per International Myeloma Working Group (IMWG) criteria (documented PD during/within 60 days from last dose of prior antimyeloma regimen). The treatment regimen has been described previously [8] and is shown in Fig. 1. Eligible patients underwent 3 days of lymphodepletion (cyclophosphamide 300 mg/m^2^/day and fludarabine 30 mg/m^2^/day) prior to infusion with ide-cel at the highest dose of 450 × 10^6^ CAR+ T cells. Bridging therapy was allowed after leukapheresis, provided the patient was treated with a therapy they had already been exposed to, the last dose was received  ≥ 14 days prior to lymphodepletion, and baseline disease staging was repeated after bridging therapy.

### Endpoints and assessments

The primary endpoint was ORR, defined as the proportion of patients with a best overall response (BOR) of at least partial response (PR), and was based on response assessments in the treated population. The key secondary endpoint was CR rate (defined as the proportion of treated patients with a BOR of CR or stringent CR [sCR]). All responses were based on the IMWG criteria [15] and adjudicated by the independent review committee. Other secondary endpoints included duration of response (DOR; defined as the time from first documentation of response [≥PR] to first documentation of PD or death from any cause, whichever occurred first), PFS (defined as the time from the first ide-cel infusion to first documentation of PD, or death due to any cause, whichever occurred first), OS (defined as the time from first ide-cel infusion to death due to any cause), rate of minimal residual disease (MRD) negativity, and safety. MRD status was assessed at baseline, then at 1, 3, 6 and 12 months post-infusion. Exploratory endpoints included characterization of the expansion of CAR T cells in the bone marrow and levels of soluble BCMA (sBCMA) from the serum of peripheral blood samples taken at baseline through to follow-up.

### Statistical analyses

A detailed overview of the statistical analyses can be found by Munshi et al. [8]. Efficacy analyses were conducted in the ide-cel treated population. The ORR was tested against the null hypothesis, which was an ORR of ≤ 50% at a one-sided alpha level of 0.025. If the ORR was significant, the CR rate was subsequently tested against the null hypothesis, which was a CR rate of  ≤ 10% at a one-sided alpha level of 0.025. The analysis of non-Japanese ide-cel–treated patients was performed to test the null hypotheses of ORR and CR when all non-Japanese ide-cel-treated patients had completed sufficient follow-up. If the results were positive, the same analyses were performed at the same alpha level for all patients, including Japanese patients 3 months after the last Japanese patient had been infused with ide-cel. The subgroup analysis of the Japanese patients was performed in a descriptive manner and no hypothesis testing was conducted. DOR, PFS, and OS were estimated using Kaplan–Meier curves. Safety was summarized descriptively.

### Ethics and study oversight

All patients provided written informed consent in accordance with the Declaration of Helsinki and local guidelines before study entry. The study received approval from institutional review boards or independent ethics committees at each site prior to study initiation and was carried out in accordance with applicable national, state, and local laws.

## Results

### Patient population and disposition

In the Japanese sub-population, 10 patients were screened across 4 study sites, with 9 meeting the eligibility criteria. One patient was excluded as the patient had a known central nervous system involvement with myeloma. All 9 patients underwent leukapheresis and were treated with ide-cel at the highest target dose of 450 × 10^6^ CAR+ T cells (Fig. 1). The median (range) time from leukapheresis to infusion was 47 days (41–61), from leukapheresis to ide-cel product release was 27 days (25–41), and from product release to ide-cel administration was 18 days (12–32). Eight patients (89%) received bridging therapy before infusion. At the time of data cutoff (December 21, 2020; Fig. 1), 7 patients remained in the study and 2 patients had died.

Baseline characteristics are shown in Table 1. The median (range) age was 54 (38–73) years and the median (range) time since diagnosis was 3.6 (1.0–7.9) years. The median (range) number of prior antimyeloma regimens was 4 (3–15). In terms of disease characteristics at baseline, 22% of patients had revised International Staging System stage III disease, 33% had high tumor burden (≥ 50% CD138+ plasma cells in the bone marrow), 67% had ≥ 50% tumor BCMA expression, 56% had extramedullary disease, and 22% had high-risk cytogenetics (including del[17p] and *t* [4;14]).

**Table 1:** Baseline patient characteristics

Characteristic	Japan (*n* = 9)
Age, median (range), years	54 (38–73)
Male sex, *n* (%)	7 (78)
Weight, median (range), kg	68 (49–89)
ECOG performance status, %	
0	67
1	33
2	0
R-ISS stage,^a^ %	
I	22
II	44
III	22
High-risk cytogenetics,^b^ *n* (%)	2 (22)
High tumor burden,^c^ *n* (%)	3 (33)
Tumor BCMA expression ≥ 50%,^d^ *n* (%)	6 (67)
Bone marrow aspirate plasma cells, median (range), %	4 (0–95)
Extramedullary disease, *n* (%)	5 (56)
Soluble BCMA, median (range), ng/mL	136 (14–593)
Lymphocyte count, median (range), 10^9^/L	1.10 (0.19–2.11)
Time since initial diagnosis, median (range), years	3.6 (1.0–7.9)
Prior antimyeloma regimens, median (range)	4 (3–15)
Prior autologous SCT, %	
Any	78
> 1	22
Bridging therapy for multiple myeloma, *n* (%)	8 (89)
Corticosteroids	8 (89)
Immunomodulatory agents	6 (67)
PIs	4 (44)
Monoclonal antibodies	2 (22)
Refractory status, *n* (%)	
Anti-CD38 antibody–refractory	6 (67)
Triple-refractory^e^	3 (33)
Penta-refractory^f^	0

*BCMA* B-cell maturation antigen, *ECOG* Eastern Cooperative Oncology Group, *PI* proteasome inhibitor, *R-ISS* revised International Staging System, *SCT* stem cell transplantation

^a^One patient in Japan had unknown R-ISS stage

^b^Includes del(17p) and t(4;14)

^c^Defined as  ≥ 50% CD138+ plasma cells in bone marrow[21]

^d^No minimum tumor BCMA expression was required for study entry

^e^Refractory to an immunomodulatory agent, PI and anti-CD38 antibody

^f^Refractory to lenalidomide, pomalidomide, bortezomib, carfilzomib, and daratumumab

### Efficacy

**Fig. 2:**
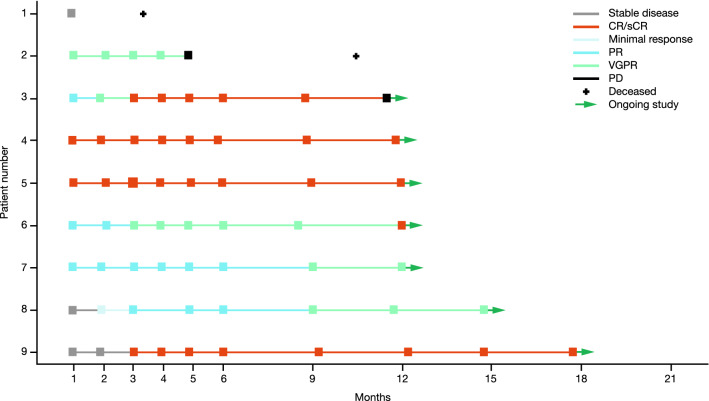
Kinetics of response. The response assessments in the treated population were according to the IMWG criteria and adjudicated by the independent review committee. A square indicates when the response was recorded. *CR/sCR* complete response/stringent complete response, *PD* progressive disease, *PR* partial response, *VGPR* very good partial response

At a median (range) follow-up of 12.9 months (3.3–17.8), in the 9 patients treated with ide-cel, the ORR was 89% (*n *= 8) (Fig. 2 and Table 2). Five patients (56%) achieved a sCR and 3 (33%) a very good PR (VGPR). The remaining 1 patient had a BOR of stable disease (SD). Of the 5 patients with a sCR, 4 had extramedullary disease (which resolved after ide-cel infusion) and 1 had high-risk cytogenetics at baseline; 1 patient with a VGPR had both extramedullary disease (non-measurable lesions at baseline and remained non-measurable after ide-cel infusion) and high-risk cytogenetics at baseline. Of the 9 patients treated with ide-cel, 6 were evaluable for MRD status; all 6 were MRD negative at month 1, 5 at month 3, 4 at month 6, and 1 at month 12.

**Table 2:** Efficacy

	Japan (*n *= 9)
BOR, *n* (%)	
Stringent complete response	5 (56)
Complete response	0
Very good partial response	3 (33)
Partial response	0
Stable disease	1 (11)
Progressive disease	0
Not evaluable	0
DOR,^a^ median (range), months	NR (3.9–NR)
PFS, median (range), months	NR (4.9–NR)
OS, median (range), months	NR (3.3–NR)
6 months (%)	89
1 year (%)	78

*DOR* duration of response, *NR* not reached, *OS* overall survival, *PFS* progression-free survival

^a^Only patients with a response of  ≥ PR were included in the analysis

In patients with a BOR of  ≥ PR, the median (range) time to response was 1.0 month (1.0–3.1), and in patients with a BOR of ≥ CR the median (range) time to ≥ CR was 3.1 months (1.0–12.0). Two patients with an initial response of PR later achieved sCR by month 3 and month 12, respectively. One patient with an initial response of SD also achieved sCR by month 3. Median (95% CI) DOR was not reached (NR) (3.9 months–NR) (Table 2 and Fig. S1). At the time of data cutoff, 6 of the 8 patients with  ≥ PR and 4 of the 5 patients with ≥ CR had an ongoing response.

The median PFS (95% confidence interval [CI]) was NR (4.9 months–NR), and the 6 month and 12 month Kaplan–Meier estimates of PFS rates were 88% and 75%, respectively (Table 2 and Fig. S2). The median OS (95% CI) was also NR (3.3 months–NR), and the 6 month and 12 month Kaplan–Meier estimates of OS rates were 89% and 78%, respectively (Table 2 and Fig. S3).

### Safety

Adverse events of special interest occurred in all patients in the Japanese cohort (Table 3). All patients experienced grade 3/4 cytopenia, including neutropenia (100%), leukopenia (89%), lymphopenia (78%), thrombocytopenia (67%), and anemia (56%), within 8 weeks of infusion. Most of these events were related to the lymphodepleting chemotherapy administered before ide-cel infusion. Six patients experienced persistent grade 3/4 neutropenia within 1 month of infusion, and the median (range) time to recovery was 1.9 (1.9–8.6) months. Six patients experienced persistent grade 3/4 thrombocytopenia within 1 month of infusion, with a median (range) time to recovery of 1.9 (1.2–9.3) months in 5 patients; the remaining 1 patient died due to unrelated causes before the thrombocytopenia was resolved. Late-onset neutropenia (first reported between 8 weeks and 24 months after infusion) was observed in 3 patients (1 at grade 3 and 2 at grade 4).

**Table 3:** All-cause adverse events of special interest

	Japan (*n* = 9)	
	Any grade	Grade 3/4
Adverse events of special interest, *n* (%)	9 (100)	9 (100)
Cytopenia	9 (100)	9 (100)
Neutropenia	9 (100)	9 (100)
Leukopenia	8 (89)	8 (89)
Lymphopenia	7 (78)	7 (78)
Thrombocytopenia	6 (67)	6 (67)
Anemia	5 (56)	5 (56)
Febrile neutropenia	1 (11)	1 (11)
Cytokine release syndrome	9 (100)	0
Infections	5 (56)	1 (11)
Influenza	2 (22)	1 (11)
Cytomegalovirus infection	1 (11)	0
Otitis externa	1 (11)	0
Upper respiratory tract infection	1 (11)	0
Neurotoxicity	2 (22)	0
Disorientation	1 (11)	0
Headache	1 (11)	0
Macrophage activation syndrome	1 (11)	0
Hemophagocytic lymphohistiocytosis	1 (11)	0

All patients in the Japanese cohort experienced  ≥ 1 CRS event (maximum grade 2), with all events occurring within the first 8 weeks after ide-cel infusion (Table 4). Grade 1 CRS occurred in 4 patients (44%) and grade 2 in 5 patients (56%). The median (range) time to onset of CRS was 1 (1–2) days and the median (range) duration was 6 (3–12) days. The median (range) time to onset of grade 2 CRS was 2 (1–3) days. CRS events were well resolved: 6 patients (67%) received 1 dose of tocilizumab, 2 (22%) received > 1 dose of tocilizumab, and 3 (33%) received steroids. A patient who experienced grade 2 CRS on day 1 developed grade 2 macrophage activation syndrome (MAS) on day 2 post-infusion; the CRS event resolved on day 7 but the MAS lasted until day 22.

**Table 4:** CRS and neurotoxicity

	Japan (*n *= 9)
≥ 1 CRS event, *n* (%)	9 (100)
Maximum grade,^a^ *n* (%)	
1	4 (44)
2	5 (56)
3	0
Onset, median (range), days	1 (1–2)
Duration, median (range), days	6 (3–12)
Patients receiving tocilizumab, *n* (%)	8 (89)
1 dose	6 (67)
> 1 dose	2 (22)
Patients receiving steroids, *n* (%)	3 (33)
≥ 1 neurotoxicity event, *n* (%)	1 (11)
Maximum grade,^b^ *n* (%)	
1	0
2	1 (11)
3	0
Onset, days	4
Duration, days	19
Patients receiving tocilizumab, *n* (%)	0
1 dose	0
Patients receiving steroids, *n* (%)	1 (11)

*CRS* cytokine release syndrome

^a^CRS was graded per Lee criteria

^b^Neurotoxicity was graded per NCI CTCAE v4.03

One patient experienced a grade 2 neurotoxicity event (Table 4), with a time to onset of 4 days and a duration of 19 days. The neurotoxicity event in this patient was well controlled with steroids.

In total, 2 patients died during the study period, 1 from plasma cell myeloma and 1 due to general health deterioration.

### Cellular kinetics

**Fig. 3:**
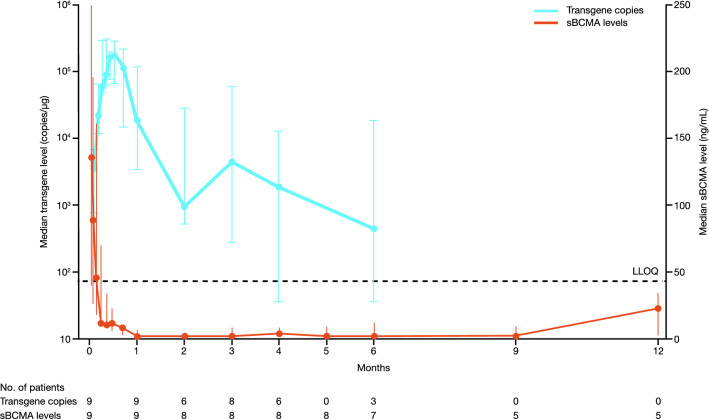
Cellular kinetics. Data cutoff for transgene data was April 7, 2020. *sBCMA* soluble B-cell maturation antigen

Cellular kinetics are shown in Figure 3. The median (range) *C*_max_, AUC_0–28_, and *T*_max_ for transgenes were 297,584 (49,887–1,236,332) copies/µg, 3,712,583 (512,838–18,438,020) copies*days/µg, and 11 (7–14) days, respectively. sBCMA levels decreased rapidly after infusion, and this decrease corresponded with an increase in transgene levels (Fig. 3). CAR T cells were detected in 7 of 8 patients tested at 3 months and 2 of 3 patients tested at 6 months. In addition, anti-drug antibody was detected in 5 patients who achieved a response (sCR, *n* = 3; VGPR, *n* = 2), with cellular expansion also observed in all of these patients.

## Discussion

This sub-analysis of KarMMa is one of the first studies, along with the sub-analysis of CARTITUDE-1 [16], to evaluate the safety and efficacy profile of a CAR T cell therapy targeting BCMA in Japanese patients with RRMM. All Japanese patients who underwent leukapheresis successfully received ide-cel infusion. Ide-cel at a target dose of 450 × 10^6^ cells demonstrated deep and durable responses, with approximately half of the patients achieving a sCR. The safety profile was consistent with that of the non-Japanese patient population.

Based on the results from all patients in the KarMMa trial [8], the highest dose of ide-cel (450 × 10^6^ cells) was selected for the treatment of all patients in the Japanese cohort. At a median follow-up of 12.9 months, the ORR was 89% and the CR rate was 56%. All patients who were evaluable for MRD were MRD-negative, demonstrating a deep response. Consistent with the non-Japanese population in the KarMMa trial and other trials with CAR T cell therapy [7, 17], some patients with an initial response of PR or SD later achieved CR. Also, responses were observed across patients with multiple high-risk features (extramedullary disease/high-risk cytogenetics). Per the efficacy criteria, of the 5 patients who achieved a sCR, 4 had extramedullary disease at baseline but the condition later resolved. At data cutoff, 6 of 8 patients (4 of 5 patients with CR) maintained an ongoing response, suggesting a durable response with ide-cel in the Japanese population. One patient in the Japanese cohort experienced PD in the 1-year follow-up after achieving sCR during months 3–9. At the time of data cutoff, only 2 patients had died in the Japanese population.

Overall, the results observed in the Japanese cohort were consistent with the non-Japanese cohort, where the null hypothesis (≤ 10% of patients receiving ide-cel would have a BOR of CR/sCR) was rejected, although comparisons between the Japanese and non-Japanese cohorts should be interpreted with caution due to the small sample size for the Japanese cohort. A numerically higher ORR was observed in the Japanese population (89%) than in the non-Japanese population (73%) [8]. Moreover, the PFS rate at 12 months was higher in the Japanese population than in the non-Japanese population (75% and 52%, respectively). These differences may be due to variations in patient background; for example, the Japanese cohort was younger, had a lower tumor burden, Eastern Cooperative Oncology Group performance status, and baseline sBCMA levels, and had fewer prior antimyeloma regimens than the non-Japanese cohort. The patients in the Japanese cohort also had a higher median lymphocyte count than the non-Japanese cohort and no patients receiving alkylating agents as their last therapy, which may partly account for the differences, as previously, median absolute lymphocyte count and longer time since exposure to alkylating agents were associated with better outcomes in patients with RRMM treated with ide-cel [18]. The small sample size of the Japanese cohort, however, does not allow for accurate comparisons and may also account for some of these differences.

The overall safety profile in the Japanese cohort was predictable, with early onset and resolution of CRS and neurotoxicity consistent with the non-Japanese population [9] and the previous report from the KarMMa study [8], and no new safety concerns were identified. All patients experienced grade 1/2 CRS, with a median onset of 1 day after administration, and events were well managed with tocilizumab or steroids. Early-onset CRS that occurs within a predictably short timeframe is one of the characteristics of ide-cel, with all CRS events in the Japanese population occurring within a maximum of 2 days after infusion. From recent studies, other CAR T cell therapies are associated with a relatively longer time to onset, such as axicabtagene ciloleucel (median [range] time to onset of 2 [1–12] days [17]), lisocabtagene maraleucel (time to onset of 5 days [range 1–14] [19]) and ciltacabtagene autoleucel (cilta-cel; median [interquartile range] time to onset of 7 [5–8] days [7]). The lower number of infused cells with cilta-cel (target dose 0.75 × 10^6^ cells/kg [7]) compared with ide-cel (target does of 450 × 10^6^ cells) and the differences in time to peak expansion (median [range] of 13 [9–210] days with cilta-cel [20] and 11 [7–14] days with ide-cel) may in part explain the differences in onset of CRS.

Neurotoxicity after ide-cel infusion was infrequent in the Japanese cohort. Only 1 patient experienced grade 2 neurotoxicity, and this was well managed. Potential factors that could have triggered the neurotoxic event in this patient include grade 2 CRS prior to and overlapping with neurotoxicity, extramedullary disease experienced between screening and baseline, and possible rapid disease progression that occurred during bridging therapy with lenalidomide, carfilzomib, and dexamethasone. However, there does not appear to be an association of peak CAR T cell or lactate dehydrogenase levels with neurotoxicity in this patient as they did not have higher peak levels of CAR T cells or lactate dehydrogenase levels than patients who did not experience neurotoxicity. The rate of neurotoxicity in the Japanese cohort is consistent with the low rates of neurotoxicity seen in the non-Japanese cohort: 18% of the patients experienced any-grade neurotoxicity.

The cellular kinetics findings were similar to previous reports from the non-Japanese patient population in the KarMMa trial [8], with cell expansion and a rapid decrease in sBCMA observed after ide-cel infusion. Post-infusion anti-drug antibody was detected in 5 patients and, in alignment with previous results, did not affect response or cell expansion in these patients.

Across all KarMMa analyses, patients with RRMM treated with ide-cel showed deep and durable responses and superior responses compared with non-CAR T cell therapies [14]. In addition, unlike immunomodulatory drugs, PIs, and anti-CD38 antibodies, CAR T cell therapy is a promising treatment option that could offer a treatment-free period in this difficult-to-treat population. However, there are currently no long-term data surrounding the durability of a response with ide-cel or other CAR T cell therapies, and further research is required to produce a functional cure for the majority of patients with RRMM.

This study had several limitations. With only 9 patients in the Japanese cohort, the sample size was small, therefore comparisons must be made with caution. The study was also a single-arm, open-label study; therefore, no direct comparisons can be made to other therapies or populations.

In this sub-analysis of the KarMMa study, Japanese patients treated with ide-cel had an ORR of 89% and a CR rate of 56%, with a median follow-up of 12.9 months. CRS and neurotoxicity events were well managed and resolved within a short period of time. These results support ide-cel as a treatment option for heavily pretreated Japanese patients with RRMM and suggest a favorable clinical benefit–risk profile.

## Supplementary Information

Below is the link to the electronic supplementary material.

Supplementary file1 (DOCX 468 KB)

## Data Availability

The Bristol Myers Squibb policy on data sharing may be found at https://www.bms.com/researchers-and-partners/independent-research/data-sharing-request-process.html.
