# Supplementary material for: Phase 2 results of idecabtagene vicleucel (ide-cel, bb2121) in Japanese patients with relapsed and refractory multiple myeloma
Source: Int J Hematol. 2023 Jan 24;117(5):729–37. doi: 10.1007/s12185-023-03538-6 (PMC10121508; doi:10.1007/s12185-023-03538-6)
Supplement: Supplementary file 1 — Supplementary file1 (DOCX 468 KB) [file 12185_2023_3538_MOESM1_ESM.docx]

# Supplementary Material

### Fig. S1 Duration of response


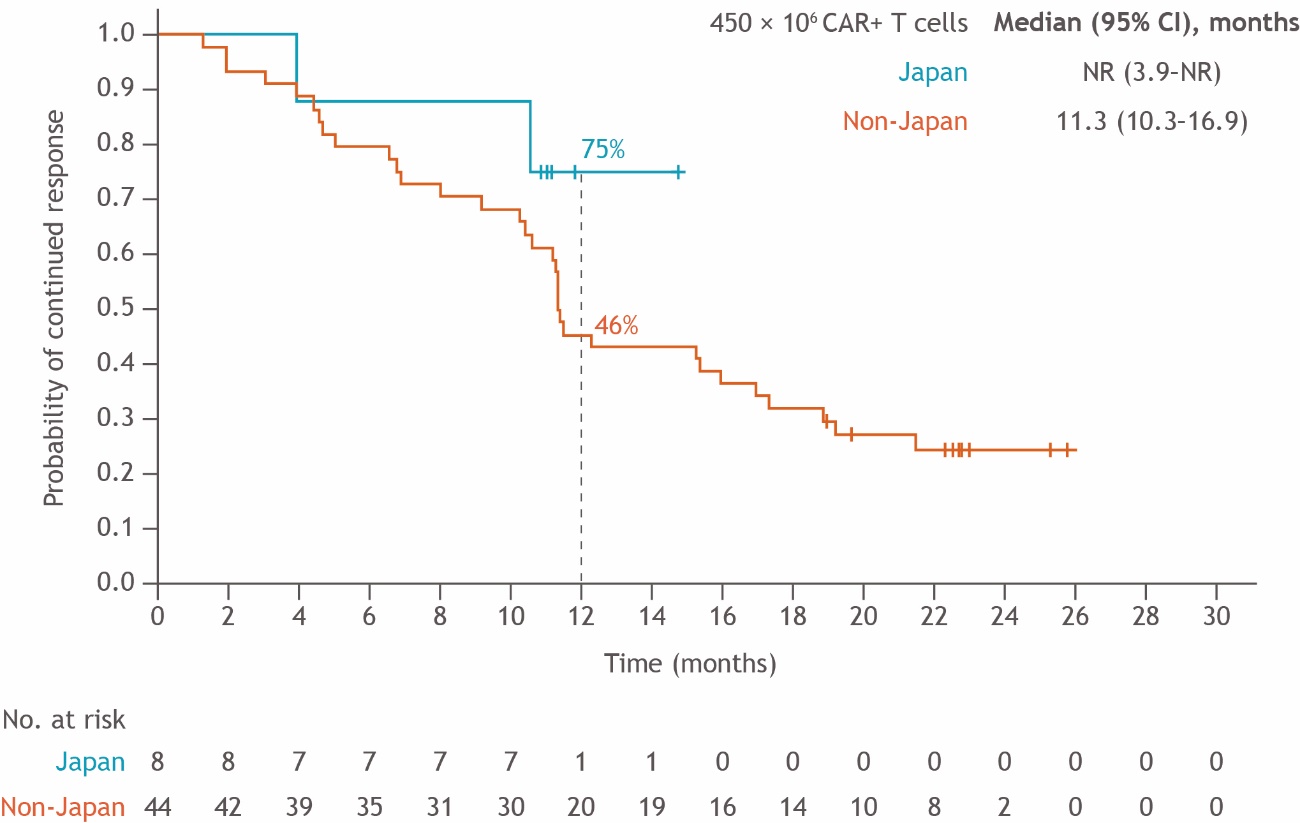


*CAR* chimeric antigen receptor, *CI* confidence interval, *NR* not reached

### Fig. S2 Progression-free survival


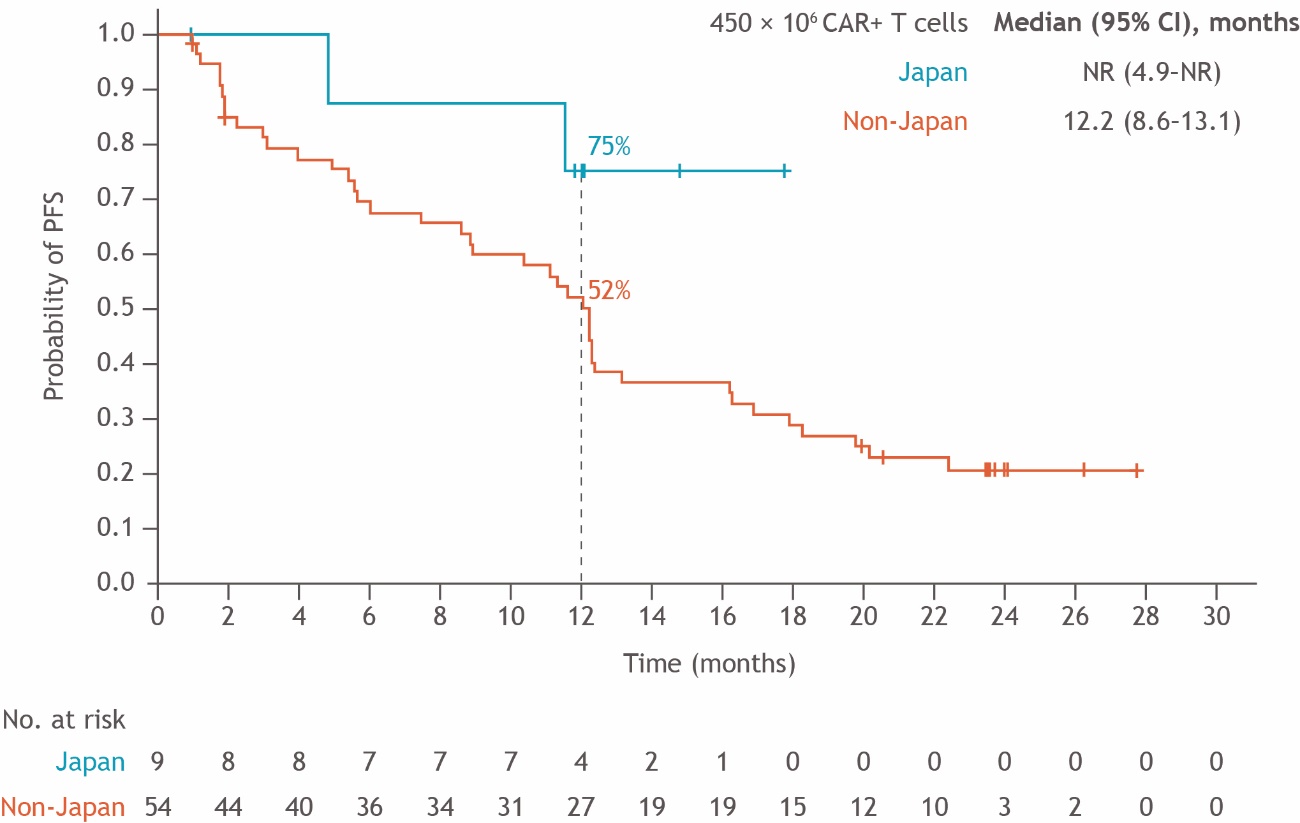


*CAR* chimeric antigen receptor, *CI* confidence interval, *NR* not reached

### Fig. S3 Overall survival


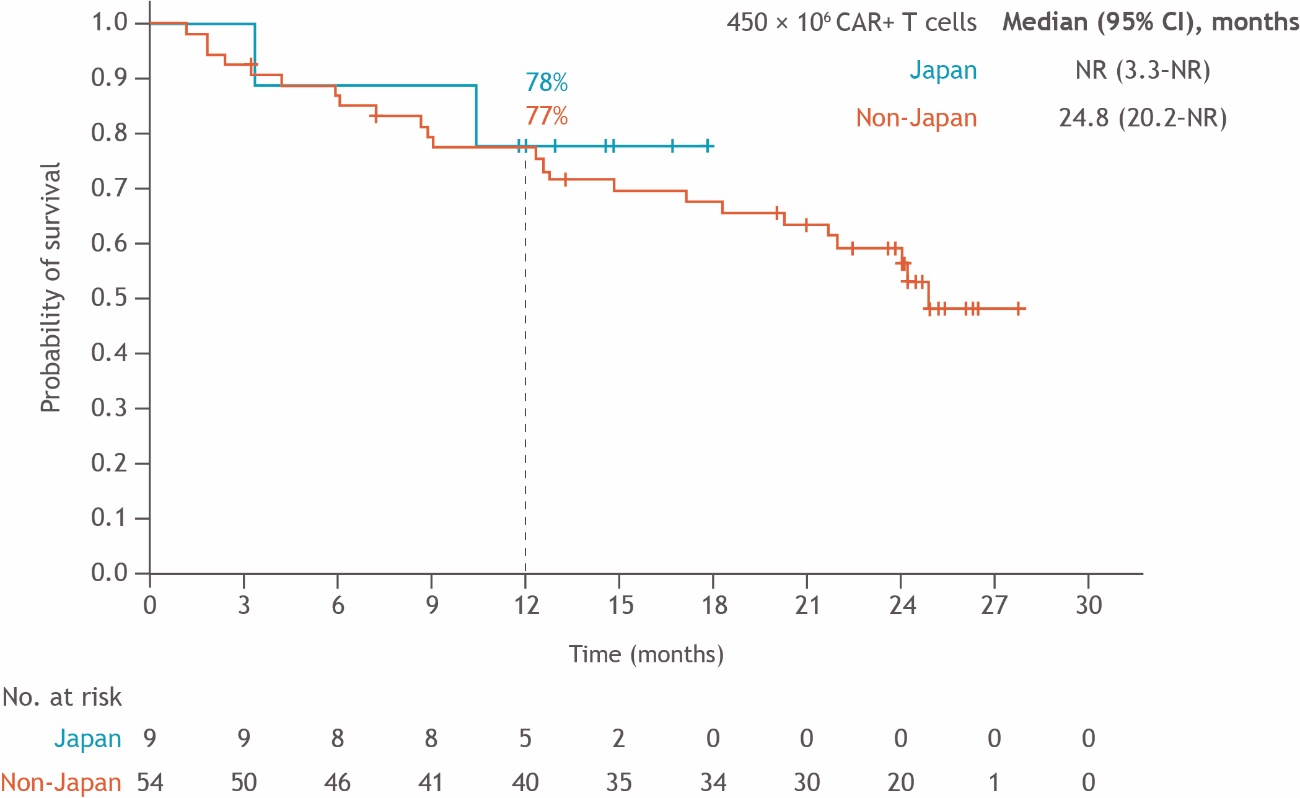


*CAR* chimeric antigen receptor, *CI* confidence interval, *NR* not reached
